# Gaps in the implementation of COVID-19 mitigation measures could lead to development of new strains of antimicrobial resistant pathogens: Nigerian perspective

**DOI:** 10.11604/pamj.2021.40.12.23274

**Published:** 2021-09-03

**Authors:** Ibrahim Yusuf, Faruk Sarkinfada

**Affiliations:** 1 Department of Microbiology, Faculty of Life Sciences, College of Natural and Pharmaceutical Sciences, Bayero University Kano, Kano, Nigeria,; 2 Department of Health and Medical Sciences, Khawarizmi International College, Abu Dhabi, United Arab Emirates

**Keywords:** Antibiotic resistance, COVID-19, antimicrobial abuse, lockdown, Nigeria

## Abstract

The severe acute respiratory syndrome corona virus 2 (SARS-CoV-2) is a new virus that is responsible for COVID-19, a disease that complicate health conditions and results in death. The total diversion of attention of government and health care workers (HCWs) to prevent the escalation of the pandemic disease has placed a great barrier to diagnosis and treatment of other illnesses that share common symptoms with COVID-19, and that has consequently enabled the endemic practice of self-antimicrobial medication to increase in Nigeria. Development of secondary infections in COVID-19 and in other conditions, caused by antibiotic resistant pathogens could make them more deadly now or in the future. The mitigation strategies adopted in Nigeria and its States, which include enforcing social distancing, partial or total lockdown, and restricting access to health care facilities for non COVID-19 patients, have further increased the demand of antimicrobial agents from unauthorized outlets in communities for inappropriate use. A cross-sectional survey of 162 randomly selected individuals that visited medical stores and 170 medical store owners to evaluates the level of self-medication with five oral broad spectrum antibiotics and antimalaria during the lockdown revealed an increase (68.5%) in practice of self-medication with at least one of the antimicrobial and emergence of new abusers. Blind treatment of symptoms of malaria and common cold without diagnosis and health care consultation was nearly 100%. Irrational use of sanitizers, disinfectants and other cidal agents that can fuel antimicrobial resistance has drastically increased in communities. Exposure of microorganisms in the environment without caution to large volume of fumigants is increasing on daily basis. We strongly recommend that while mitigating SARS-CoV-2 virus spread, efficacious and feasible technological, social, economic and behavioral interventions that will also control the evolution and spread antimicrobial resistant microorganisms should be applied.

## Commentary

The severe acute respiratory syndrome corona virus 2 (SARS-CoV-2) is a new virus that causes health complications and overwhelm health care facilities. The novel virus which was discovered in Wuhan, China in 2019 was declared pandemic by WHO on March 11, 2020 [1] and since then it has progressively claimed thousands of lives and grounded economic activities globally. Its nature or style of transmission from one person to another and lack of vaccine and drugs for prevention and treatment has made all governments, international institutions and organizations, the healthcare workers (doctors, nurses, laboratory scientists), the private sector, financial bodies, civil society and the general public to focus their entire attention on how to contain the virus. Nigeria reported its index case of COVID-19 on February 27, 2020 and the number of confirmed cases and deaths has continued to rise on daily basis [2]. The total diversion of attention to prevent the escalation of the pandemic situation has placed a great barrier to diagnosis and treatment of other illnesses that share common symptoms with COVID-19, and that has consequently enabled the endemic practice of self-antimicrobial medication to increase in the country.

Focusing on antibiotics during a viral pandemic might seem strange, but secondary infections caused by antibiotic resistant pathogens (ARPs) are what could make COVID-19 and other illnesses more deadly. Development and rise of ARPs in Nigeria is suggested to be anthropogenically driven by exposure of microbes to unnecessary antimicrobial and other chemical agents in hospitals, environments and communities [3]. Further exposure of microbes to these agents has gone up rapidly during the pandemic following the new practices adopted by individuals and state government to contain the virus which could further expose the community members to the evolution of more deadly ARPs. As community transmission of COVID-19 in Nigeria was confirmed by the Nigeria Ministry of Health and the Nigeria Center for Disease Control (NCDC) [4], raised anxieties among community members in fear of being labeled as suspected COVID-19 patient and consequently being recruited into isolation and treatment centers, forced people to quickly self-treat symptoms that are consistent with COVID 19 with over or under dosage of available genuine and counterfeit over-the-counter antimicrobial agents. The mitigation measures adopted in the country and in some States, which include enforcing social distancing, partial or total lockdown, and restricting access to health care facilities for non COVID-19 patients, have further raised the demand of antimicrobial agents from unauthorized outlets in communities. The burden was much higher in residential areas and rural communities where pockets of medicine stores are still providing full or skeletal services.

We conducted a cross-sectional survey of 162 randomly selected individuals that visited medical stores and 170 medical store owners to evaluates the level of self-medication with five oral broad spectrum antibiotics (ciprofloxacin, ampicillin-cloxacillin, amoxicillin, co-trimoxazole and tetracycline) and antimalaria drugs (artemicinin combination therapy, chloroquine, sulfadoxine-pyrimethamine) which are commonly used to treat respiratory symptoms and fever (especially malaria) during the lockdown in a state with more than 15 million people. Of the total participants, about 62% claimed to have fever and 26% had respiratory symptoms in the last 24 hours. The practice of self-medication with at least one of the antimicrobial was 68.5%. Blind treatment of symptoms of malaria and common cold without diagnosis and health care consultation was practiced by nearly 66% of the respondents before the pandemic. The most used anti-microbials for self-medication were antimalaria (especially ACT) followed by broad spectrum antibiotics (in decreasing order), co-trimoxazole, amoxicillin, ciprofloxacin, ampicillin-cloxacillin, and tetracycline. It was not possible to quantify the amount of each antimicrobial agent consumed in each shop per community during the lockdown. However, the rate was high enough to have caused shortage of many of the antimicrobials in the medical stores studied, which could further render them less effective in future use. Fear of facing the risk of health deterioration due to restrictions during the lockdown, has caused 35.8% of the participants who have never practiced self- medication to practice self-medication and more individuals to consult unqualified personnel for prescription. The risk of contracting SARS-CoV-2 by those having fever and respiratory symptoms and who abuse antimicrobial was very high as maintaining social distancing and wearing face mask was observed only 12.4% of them. The situation of antimicrobial abuse could be worse in rural communities where previous study have indicated rise in self-antibiotic treatment and most of the bacteria commonly isolated from clinical samples in the communities were already resistant to many classes of antibiotics including imipenem that was not freely available [5]. Even though misuse of antimicrobials through failing to complete a course of antibiotics, or ignoring daily dose intervals can both increase the chance of commonly isolated pathogens in the community to survive (i.e. develop resistance) and multiply, but economic hardship has forced 41.9% of the respondents to use under dose of the antibiotics purchased against their wish (Table 1).

**Table 1:** assessment of antimicrobial usage and practice of mitigation practice by antimicrobial buyers during one week COVID-19 pandemic lockdown

Parameter/Questions	Number of response (%)
**Age**	
Average (STD)	19.5 (3.0)
Range (years)	15-56
**Sex**	
Male (%)	84 (51.9)
Female (%)	78 (48.1)
**Purported symptoms of illness**	
Fever (%)	100(61.7)
Respiratory symptoms (%)	43(26.5)
Others (%)	19 (11.7)
**Blind treatment with antimicrobials**	
Practiced before COVID-19 pandemic (%)	104 (64.2)
Not practiced before COVID-19 pandemic (%)	58 (35.8)
**Type of antimicrobial self-used during lockdown**	
Ampicillin-cloxacillin (%)	11 (6.7)
Amoxicillin	23(14.1)
Ciprofloxacin	21(12.9)
Tetracycline	11(6.7)
Co-trimoxazole	26 (16.0)
Arthemicin Combination therapy	64 (39.5)
Chloroquine	06 (3.7)
**Dosage**	
Incomplete dosage	96 (59.3)
Complete dosage	66 (40.7)
**Reason for incomplete dosage**	
Financial constraint	28 (42.4)
Non-availability of drugs	6(9.0)
Enough to treat the symptoms	17(25.7)
Don’t know	15 (22.7)
**Mitigation strategy employed?**	
Wear of Face mask	12 (7.4)
Social distancing	06 (3.7)
Use of hand sanitizer	02 (1.2)
None	142 (87.6)
**Knowledge of risk of acquiring Covd-19**	
Yes (%)	92 (56.7)
No (%)	59(36.4)
Not interested (%)	11(6.7)

It was not clear to what extent COVID 19 patients at treatment facilities were being exposed to antimicrobial agents in Nigeria. However, combination of antimalaria drug (hydroxychloroquine) and azithromycin with some other antiviral agents has been a popular treatment option for them [6]. Few studies have reported how antibiotics used in treating secondary infections in COVID-19 infections has increases the level of antibiotic resistance in COVID-19 patients [7]. In addition to antimicrobial agents, persistent use of disinfectants, antiseptics and other cleaning agents to disinfect or sanitize surfaces has an unintended effect of fueling the evolution of ARPs. Microbes especially bacteria can acquire resistance following inappropriate or excessive use of these cidal agents. Under usage through over dilution of these chemical agents or over usage through over concentrating or intermittent use can provide a survival chance to the resistant strains. Use of sub optimal concentration of disinfectants (i.e. concentration that is not enough to eliminate microorganisms) in public places has been the practice in some markets, banks, and other public places. Scarcity and high price of disinfectants and sanitizers could have led to its sub-optimal use by dissolving required amount into a larger volume of diluents, which could drive microorganisms to develop resistance.

Extensive calls by HCWs, NCDC [2], WHO, other scientists and individual/government sponsored media campaigns on the need for improved personal hygiene practices through hand washing and use of alcohol-based hand sanitizers, has caused a significant rise in the use and sale of hand sanitizers. The surge in price and scarcity has also made it possible for substandard sanitizers to flood the market while advice about their prudent use or of the consequences of their misuse is not available. A survey of 26 hand sanitizer users revealed only 42.3% can appropriately use it. Exaggerative practice was observed in 4 out of the 26 (15.3%) respondents, who often use the sanitizers to rub their hand up to elbow and another 11.5% used it to rub their nostrils with intention of eliminating virus. Increasing (10 fold) tolerant of some bacteria to killing by alcohol based disinfectant in a study conducted by Pidot *et al*. [8] was scary. Even if the sanitizers protect against COVID-19, the possibility of developing new strains of ARPs due to excessive or persistent under use is imminent. Concern about the role environment plays in both the evolution and transmission of antibiotic resistant bacteria is still evolving in Nigeria. The sudden exposure of microorganisms in the environment (soil, water and sewage) to unknown concentration of disinfectants and fumigants used to clean markets, roads and other places during the pandemic could lead to an increase in the number of ARPs. Many Nigerian States with cases of COVID-19 have fumigated markets and other public places with the likes of chlorine disinfectant and calcium hypochlorite. Future exposure of human and animal to environmental resistant bacteria would put a greater pressure on our already stretched healthcare systems, potentially leading to more health complications and death.

## Conclusion

Individual and government enforced mitigation strategies against COVID-19 could further fuel the emergence of antibiotic resistant bacteria and genes which could easily spread among human, animals and the environment. We recommend that Nigerian government and AMR stakeholders should, while mitigating SARS-CoV-2 virus spread, apply efficacious and feasible technological, social, economic and behavioral interventions that would also control the evolution and spread antimicrobial resistant microorganisms.
